# Catalytic activity study of a laccase-like copper–gallic acid MOF and its applications in the colorimetric determination of norepinephrine and degradation of environmental pollutants†

**DOI:** 10.1039/d5ra00942a

**Published:** 2025-04-15

**Authors:** Ola G. Hussein, Yara Mohamed, Noha Mostafa, Amr M. Mahmoud

**Affiliations:** a Department of Pharmaceutical Chemistry, Faculty of Pharmacy, Future University in Egypt Cairo 11835 Egypt ola.farag@fue.edu.eg; b Department of Chemistry, School of Pharmacy, Newgiza University Km. 22 Cairo-Alex Road, Giza P. O. Box 12577 Egypt; c Department of Pharmaceutical Analytical Chemistry, Faculty of Pharmacy, Cairo University Kasr El-Aini Street ET-11562 Cairo Egypt amr.bekhet@pharma.cu.edu.eg

## Abstract

Laccases enzymes have garnered significant research interest owing to their extensive applications in pollutant degradation, the food industry, and biosensing technologies. These green biocatalysts are distinguished by the presence of four copper active sites which are integral to their enzymatic functions. Recent advancements have led to the development of copper-based organic–inorganic nanocomposites as laccase mimetics. Hence, this study focused on the synthesis and study of the catalytic properties of a copper–gallic acid metal–organic framework (Cu–GA MOF) heterostructure as a laccase mimic. Using *o*-phenylenediamine (OPD) and norepinephrine as model substrates it was observed that the synthesized Cu–GA MOF exhibited a laccase-like catalytic performance. Similar to natural enzymes and other nanozymes, Cu–GA MOF demonstrated pH-dependent catalytic activity demonstrating an optimal performance under physiological conditions. It exhibited a superior Michaelis constant (*K*_m_) of 0.06 mM, maximum reaction rate (*V*_max_) of 4.1 × 10^−3^ mM min^−1^ and superior recyclability compared with laccase at the same mass concentration. Remarkably, Cu–GA MOF displayed exceptional thermal tolerance maintaining substantial catalytic activity at temperatures up to 90 °C. In contrast to natural enzymes, Cu–GA MOF exhibited enhanced stability and recyclability underscoring its potential for diverse bio-applications. These findings highlight the promising role of Cu–GA MOF as a robust and versatile catalyst in biocatalytic and analytical applications.

## Abstract 2

Laccases enzymes have garnered significant research interest owing to their extensive applications in pollutant degradation, the food industry, and biosensing technologies.

## Graphical abstract

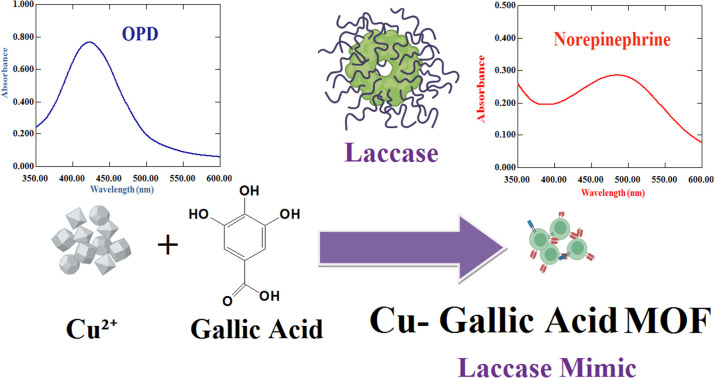

## 1. Introduction

Over the past decade, artificial nanozymes have undergone significant advancements emerging as robust alternatives to natural enzymes.^1–3^ Nanozymes bypass the inherent limitations of natural enzymes which include costly purification processes, poor stabilities, and vulnerability to environmental factors.^4–6^ A variety of nanomaterials such as metal nanoparticles, carbon-based materials, and metal oxides have been investigated for the development of nanozymes.^7–12^ These materials have demonstrated their ability to mimic various natural enzymes including superoxide dismutase, glucose oxidase, peroxidase, and catalase, with remarkable catalytic efficiencies.^13,14^ Notably, nanozymes exhibit superior catalytic activity and find extensive applications in diverse fields such as therapeutics, biosensing, and pollutant remediation.^15–17^ The strategic design and advancement of nanozymes have not only helped in overcoming the drawbacks of natural enzymes but also greatly broadened their potential applications in both biological fields and industry paving the way for innovative solutions in enzyme-based catalysis.^18,19^

Laccase is a multicopper oxidoreductase that is renowned for catalyzing the oxidation of phenolic compounds.^20–22^ In the presence of oxygen it facilitates the hydroxylation of monophenols into diphenols and their subsequent oxidation into quinones. A significant advantage of laccase is its eco-friendly nature as it neither requires nor generates toxic hydrogen peroxide during its catalytic process. Consequently, laccase has been widely employed as a green catalyst particularly in the elimination of aromatic pollutants from impure substances.^23–25^ However, despite the valuable applications of laccase its instability and high cost of purification considerably hinder its broader utilization. To address these challenges extensive efforts have been directed towards the development of laccase-mimicking materials. As the catalytic activity of laccase arises from its active sites which consist of copper and numerous copper-based organic–inorganic cysteine–histidine bridges various nanocomposites have been synthesized inspired from its intricate structure and catalytic mechanism to mimic its functions.^26,27^ Despite these advancements, the continuous development of novel laccase mimics remains a critical area of research owing to their vast potential applications across various fields.

Recently, gallic acid has emerged as a promising agent for the construction of nanozymes either with metal oxides or metals owing to its durable metal-binding affinity, multifunctionality, and excellent biocompatibility.^28,29^ Cu–gallic acid nanozymes are hybrid materials that integrate copper ions with gallic acid leveraging the strong metal-chelating ability and antioxidant properties of gallic acid. These nanozymes can mimic the catalytic activity of laccase (a multicopper oxidase) by creating a Cu^+^/Cu^2+^ redox system which is similar to the one that is found in the active sites of natural enzymes. The reductive properties of gallic acid can facilitate the reduction of Cu^2+^ to Cu^+^ thereby enhancing the catalytic efficiency. Compared with natural laccase; Cu–gallic acid nanozymes offer several advantages including excellent biocompatibility, high stability under various environmental conditions, and superior recyclability. Their eco-friendly nature, cost-effectiveness, and multifunctionality position Cu–gallic acid nanozymes as promising alternatives to traditional enzymes in biocatalytic and environmental applications.^30^

Laccase-like nanomaterials have been applied in the degradation of organic environmental pollutants including *o*-phenylenediamine (OPD) and norepinephrine (NE). These nanomaterials exhibit strong catalytic activity enabling the oxidation of such contaminants into less harmful or more biodegradable compounds. Their efficiency, stability, and eco-friendly nature make them valuable for environmental cleanup applications offering a sustainable approach to mitigating pollution.

*O*-Phenylenediamine (OPD) is a widely used industrial chemical particularly in the synthesis of dyes, polymers, and various pharmaceutical products.^31,32^ However, its release into the environment as a pollutant results in significant ecological and human health risks. OPD is classified as a toxic substance as exposure to it can lead to skin irritation, respiratory problems, and potential carcinogenic effects. Hence, its persistent presence in wastewater and industrial effluents necessitates the development of efficient environmentally friendly methods for its removal.

Previous studies have investigated the laccase-like activity of different copper-based systems such as Cu–tannic acid and Cu–cystine-aspartic acid dipeptide to enhance the enzymatic degradation of organic pollutants.^33–35^ Although these systems have demonstrated significant biocatalytic potential our research introduces a Cu–gallic acid metal–organic framework (Cu–GA MOF) system which has not been widely explored previously. The uniqueness of our study lies in its application for both the oxidation of OPD and the colorimetric determination of norepinephrine which are the areas that are not previously addressed with Cu–GA. Furthermore, our findings revealed high *V*_max_ and low *K*_m_ values of Cu–GA MOF indicating its superior catalytic efficiency and substrate affinity compared with previously reported copper-based nanozyme systems. This advancement underscores the effectiveness of Cu–GA MOF in mimicking laccase activity and its potential in environmental and analytical applications.

Laccase-like nanomaterials have also been utilized in the development of colorimetric sensors for detecting various analytes including norepinephrine (NE). These nanomaterials facilitate the direct oxidation of the target analyte using oxygen leading to the formation of a colored product. This color change enables the easy and rapid detection of the analyte making such sensors highly effective for analytical applications. Furthermore, their sensitivity, simplicity, and eco-friendly nature contribute to their growing utility in biosensing technologies.

In this study, a copper–gallic acid metal–organic framework (Cu–GA MOF) was synthesized for the purpose of emulating laccase-like catalytic activity. OPD and norepinephrine were employed as the target substrates for investigating the lacasse-like activity of the copper–gallic acid complex whereby it removed the toxic OPD from polluted environments and enabled the colorimetric determination of norepinephrine. The dual application of this biocatalytic system could provide the dual benefit of mitigating environmental contamination and facilitating the study of important biological markers. Owing to the coordination between copper ions and gallic acid, Cu–GA MOF demonstrated intrinsic laccase-mimicking activity. It also displayed remarkable catalytic stability across a wide pH range and elevated temperature conditions significantly broadening its potential applications. The findings of this study underscore the promising utility of Cu–GA MOF in biosensing, environmental remediation, and medical diagnostics.

## 2. Experimental

### 2.1. Instruments and software

Scanning electron microscopy (SEM) (Quanta FEG-250) and energy-dispersive X-ray spectroscopy (EDX) were applied to characterize the morphology of the synthesized nanoparticles. Spectrophotometric measurements were carried out using a dual-beam UV-Vis spectrophotometer (UV-1900i PC, Shimadzu Corp., Kyoto, Japan), together with UV probe software version 2.43, utilizing a 1 nm spectral bandwidth, 2800 nm min^−1^ wavelength scanning speed, and 1 cm quartz cells. The particle size of Cu–GA MOF was determined using dynamic light scattering (DLS) with a Malvern Nano-ZS zetasizer (Malvern Instruments Limited, Malvern, UK). FT-IR spectroscopy was carried out using a Shimadzu IR 435 spectrophotometer (Shimadzu Corp., Kyoto, Japan) to identify the functional groups and investigate the molecular interactions within the composite structure. Quantachrome TouchWin™ version 1.2, which is a specialized software for gas sorption and porosimetry instruments, was utilized for precise determination of surface area, pore-size distribution, and porosity. Thermogravimetric analysis (TGA) was performed using a Shimadzu TGA-50 thermogravimetric analyzer (Kyoto, Japan) to evaluate the thermal stability of Cu–GA MOF. The crystal structure of Cu–GA MOF was examined using an X-ray diffractometer (Model: Rigaku Smart Lab).

### 2.2. Materials and reagents

Gallic acid (GA), norepinephrine, and *o*-phenylenediamine (OPD) were acquired from Sigma-Aldrich (USA). Copper sulfate pentahydrate (CuSO_4_·5H_2_O), sodium hydroxide (NaOH), Tris, acetate, hydrochloric acid (HCl), and sodium dihydrogen phosphate were obtained from Bio-chem (Egypt). All the chemicals were of analytical grade and used without further purification. The phosphate buffer (pH 6.0) was prepared from a 0.02 mol L^−1^ solution of sodium dihydrogen phosphate. The pH of the solution was adjusted to 6.0 by the gradual addition of sodium hydroxide (NaOH) under continuous stirring.

### 2.3. Standard solutions

#### 2.3.1 *O*-Phenylenediamine (OPD) and norepinephrine standard solutions

Standard stock solutions of *O*-phenylenediamine (OPD) and norepinephrine each at a concentration of 1.0 mg mL^−1^ were prepared using distilled water.

## 3. Procedure

### 3.1. Synthesis of Cu–GA MOF

Synthesis of Cu–GA MOF was performed as reported in a previous study^35^ by dissolving copper sulfate pentahydrate (5 mmol) and gallic acid (5 mmol) in 100 mL of deionized water under constant stirring. The pH of the resultant mixture was adjusted to 8.0 using 0.1 M sodium hydroxide while continuously stirring for 4 h at room temperature. After completion of the reaction, Cu–gallic acid complex was collected *via* centrifugation and purified through multiple washings. The final product was dried at 60 °C under vacuum to yield Cu–GA MOF.

### 3.2. Characterization of Cu–GA MOF

The synthesized Cu–GA MOF was characterized using a combination of scanning electron microscopy (SEM), energy-dispersive X-ray spectroscopy (EDX), Fourier-transform infrared spectroscopy (FTIR), X-ray diffraction (XRD), thermogravimetric analysis (TGA), Brunauer–Emmett–Teller (BET) surface area analysis, and UV-Vis spectrometry measurements. SEM analysis was performed to provide detailed insights into the morphology, size, and shape of Cu–GA MOF. EDX was employed to analyze the elemental composition by generating elemental maps to visualize the distribution of elements offering quantitative data on the atomic percentages of each constituent element. FTIR spectroscopy was used to identify the functional groups present in the material confirming the interaction between the Cu and gallic acid. XRD analysis was conducted to determine the crystalline structure and phase composition of Cu–GA MOF. TGA was utilized to assess the thermal stability and decomposition behavior providing insights into the material's thermal properties. BET surface area analysis was carried out to evaluate the specific surface area, pore volume, and pore-size distribution for assessing the suitability of Cu–GA MOF for high-surface area applications. These comprehensive characterizations were crucial for understanding the essential properties and potential applications of Cu–GA MOF.

### 3.3. Evaluation of the laccase-like activity of Cu–GA MOF

To evaluate the catalytic activity of copper–gallic acid metal–organic framework, 100 μL of Cu–GA MOF (1 mg mL^−1^) and 100 μL of *o*-phenylenediamine (OPD) standard stock solution were mixed in 700 μL phosphate buffer solution at pH 6.0. The mixture was incubated at 40 °C for 30 minutes. Absorbance measurements were subsequently recorded using a UV-Vis spectrophotometer. A control experiment was conducted under the same conditions using 100 μL of OPD standard stock solution without Cu–GA MOF catalyst.

### 3.4. Impact of pH on the catalytic performance of Cu–GA MOF

To examine the influence of pH on the catalytic activity of Cu–GA MOF, 100 μL of (1 mg mL^−1^) aqueous solution of Cu–GA MOF and 100 μL of *o*-phenylenediamine (OPD) standard stock solution were mixed with 700 μL phosphate buffer solution and tested under a varying pH range of 4.0–9.0 using *o*-phenylenediamine (OPD) and norepinephrine as the substrates. The resulting reaction mixtures were incubated at 40 °C for 30 minutes and the absorbance values were measured using a UV-Vis spectrophotometer.

### 3.5. Impact of temperature on the catalytic performance of Cu–GA MOF

To assess the effect of temperature on the catalytic activity of Cu–GA MOF, 100 μL of aqueous solution of Cu–GA MOF (1 mg mL^−1^) and 100 μL of *o*-phenylenediamine (OPD) standard stock solution were mixed with 700 μL phosphate buffer solution at pH 6.0. The reaction mixture was incubated at varying temperatures (30 °C to 90 °C) for 30 min and the absorbance values were measured using a UV-Vis spectrophotometer. A similar procedure was conducted to evaluate the temperature tolerance of the norepinephrine standard stock solution by adjusting the pH of the phosphate buffer solution to 8.0.

### 3.6. Effect of time on norepinephrine oxidation rate

To investigate the effect of time on the oxidation rate of norepinephrine, a reaction mixture was prepared by adding 100 μL of aqueous solution of Cu–GA MOF (1 mg mL^−1^) to 100 μL of norepinephrine standard stock solution and the volume was completed using a phosphate buffer solution at pH 8.0. The samples were incubated at 40 °C for 60 minutes. Aliquots were collected at intervals of 5 minutes. The absorbance of each sample was measured using a UV-Vis spectrophotometer to monitor the oxidation process over time.

### 3.7. Norepinephrine concentration assay using Cu–GA MOF

A concentration-dependent study of norepinephrine was conducted using various volumes of norepinephrine standard stock solution (20, 30, 40, 50, 60, 70, 80, 90, and 100 μL) added to 10 mL volumetric flasks and a fixed volume of 100 μL of aqueous solution of Cu–GA MOF (1 mg mL^−1^) was added to each sample. The volume was completed using a Tris buffer at pH 8.0, obtaining final concentrations of 5.0, 10, 20, 30, 40, and 50 μg mL^−1^. The reaction mixtures were incubated at 40 °C for 30 minutes and the absorbance values were measured using a UV-Vis spectrophotometer.

### 3.8. Kinetic study of norepinephrine oxidation using Cu–GA MOF

A kinetic study of Cu–GA MOF nanozymes was performed using norepinephrine as the substrate. The catalyst concentration was maintained at 1 mg mL^−1^ while the substrate concentration was varied between 0.1 and 50 mM. The resulting data were then fitted to the Michaelis–Menten equation and the kinetic parameters (*K*_m_ and *V*_max_) were determined.

## 4. Results and discussion

### 4.1. Preparation and characterization of Cu–GA MOF

**Fig. 1:**
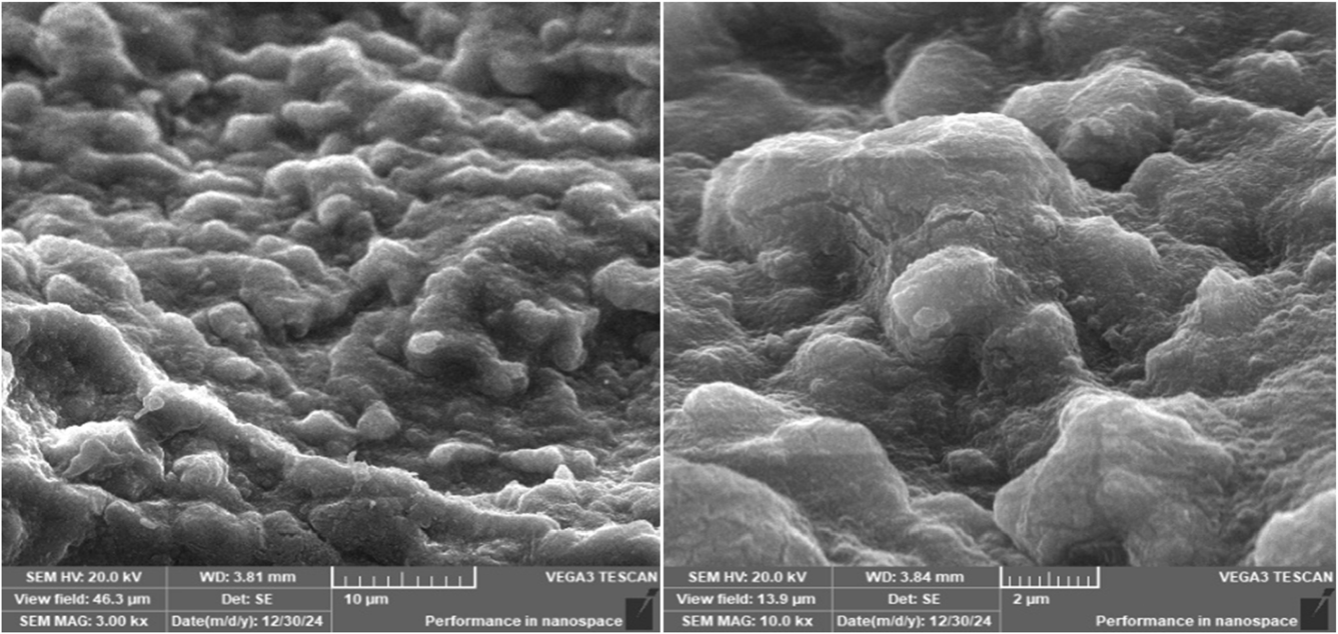
SEM images of Cu–GA MOF.

**Fig. 2:**
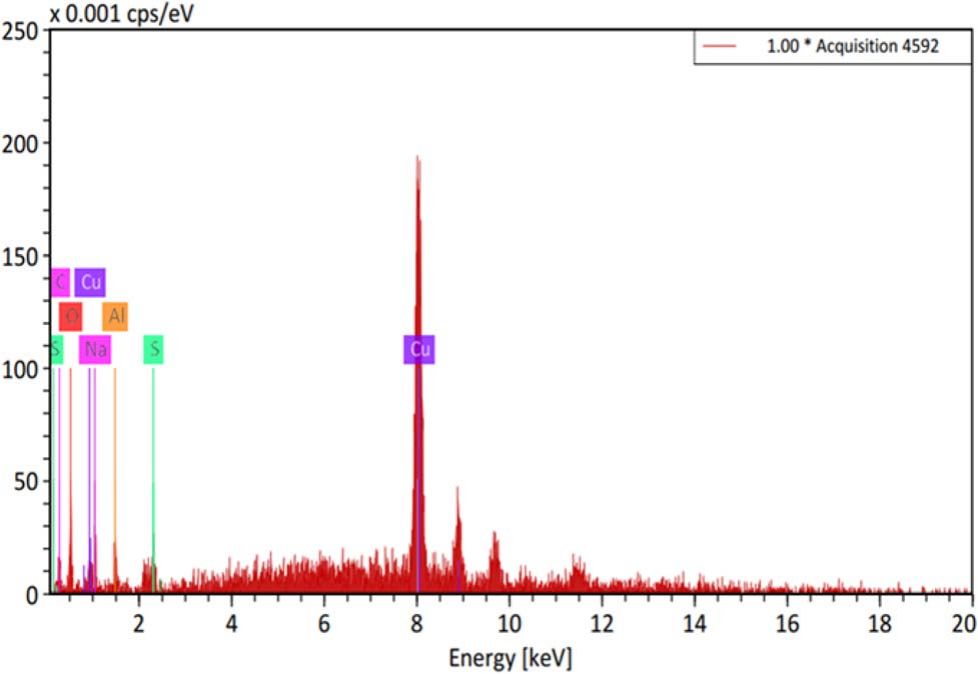
EDX spectrum of Cu–GA MOF nanocatalyst.

Synthesis of Cu–GA MOF was carried out in an aqueous medium at room temperature. Scanning electron microscopy (SEM) images revealed that the nanoparticles exhibited a sheet-like morphology indicating its uniform and well-defined structure (Fig. 1). The successful formation of Cu–GA MOF was attributed to the coordination between Cu^2+^ ions and gallic acid which was facilitated by adjusting the pH to 8.0. Energy-dispersive X-ray (EDX) analysis confirmed the presence of copper, oxygen, and carbon, validating the successful synthesis of the copper–gallic acid metal–organic framework (Fig. 2). The detection of copper in the solid phase corroborated its interaction with gallic acid supporting the formation of the desired compound. Specifically, the EDX spectra revealed distinct peaks corresponding to copper, carbon, and oxygen, with the copper peaks indicating the incorporation of Cu into the nanostructure and the carbon and oxygen peaks suggesting the presence of gallic acid in the nanoparticles.

The particle size of Cu–GA MOF was determined using dynamic light scattering (DLS) with a Malvern Nano-ZS zetasizer (Malvern Instruments Limited, Malvern, UK). The particle size was observed to be 179.5 nm with a particle distribution index (PDI) of 0.343 and zeta potential of −50.6 mV (Fig. S1†).

**Fig. 3:**
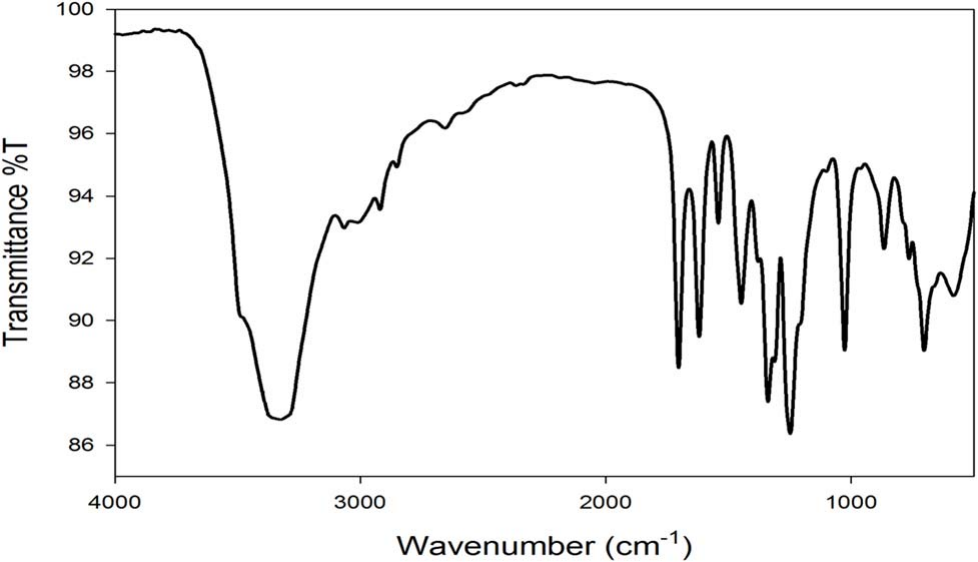
FTIR spectrum of Cu–GA MOF.

The infrared (IR) spectrum (Fig. 3) provided valuable information about the functional groups present in Cu–GA MOF. The broad absorption band observed in the 3200–3500 cm^−1^ region indicated the presence of hydroxyl (–OH) stretching vibrations. This broad peak was characteristic of the multiple hydroxyl groups in gallic acid and may shift upon complexation with Cu^2+^ owing to hydrogen bonding or coordination effects. The strong peak in the 1650–1750 cm^−1^ range corresponded to the C=O (carbonyl) stretching vibration of the carboxyl (–COOH) functional group. The 1400–1600 cm^−1^ region showed peaks associated with C=C stretching vibrations of the aromatic ring confirming the presence of the aromatic system derived from gallic acid. In the 1200–1300 cm^−1^ range, strong absorption bands appeared owing to the C–O stretching of phenolic groups which played a key role in copper coordination. Finally, the absorption band appearing in the 500–700 cm^−1^ range suggested the presence of Cu–O stretching vibrations, confirming the formation of copper–gallic acid complex through metal–ligand interactions. Overall, the IR spectrum strongly supported the coordination of Cu^2+^ ions with the carboxyl group and phenolic oxygen atoms of gallic acid leading to the formation of a stable metal–organic framework.

#### 4.1.1 Porosity analysis

The textural properties of the as-prepared mesoporous MOF were analyzed using nitrogen adsorption–desorption isotherms (Fig. S2†), and the results were summarized in Table S1.† The BET and Langmuir surface areas were determined to be 1050.40 m^2^ g^−1^ and 1480.30 m^2^ g^−1^, respectively confirming the high porosity of the sample. The BJH method yielded surface areas of 850.20 m^2^ g^−1^ (adsorption) and 825.60 m^2^ g^−1^ (desorption) while the DFT method provided a cumulative surface area of 1205.70 m^2^ g^−1^. The total pore volumes were 1.15 cc g^−1^ (BJH adsorption), 1.08 cc g^−1^ (BJH desorption), and 1.02 cc g^−1^ (DFT). Pore-size distribution analysis showed a BJH adsorption pore radius of 3.80 nm, a desorption radius of 4.10 nm, and a DFT-derived value of 3.95 nm confirming the mesoporous structure of Cu–GA MOF. The nitrogen adsorption–desorption isotherm exhibited a type IV profile with a pronounced hysteresis loop characteristic of mesoporous materials. The uniform mesopore structure of Cu–GA MOF makes it highly suitable for applications in catalysis, drug delivery, gas separation, and adsorption-based processes as the results align well with those of previously reported high-surface-area MOFs.^36–38^

**Fig. 4:**
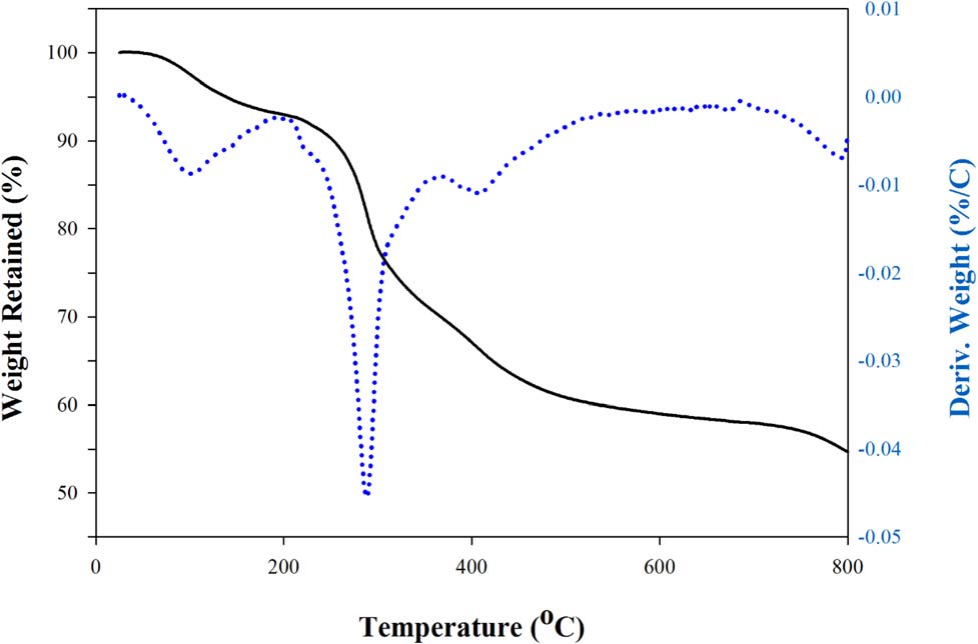
TGA analysis of Cu–gallic acid MOF.

Thermogravimetric analysis (TGA) of the synthesized Cu–GA MOF revealed a multi-step weight loss pattern indicating its thermal stability and decomposition behavior (Fig. 4). The initial weight loss observed below 150 °C corresponded to the evaporation of adsorbed moisture and residual solvents. Later, a significant weight loss occurred between approximately 200 °C and 500 °C suggesting the decomposition of the organic ligand and breakdown of the MOF structure. Beyond 500 °C, a stable residue remained likely corresponding to the formation of copper oxides (CuO or Cu_2_O). These results indicate that Cu–GA MOF exhibited good thermal stability with decomposition temperatures comparable with previously reported similar MOFs confirming the robustness of its coordination structure.^39–41^

**Fig. 5:**
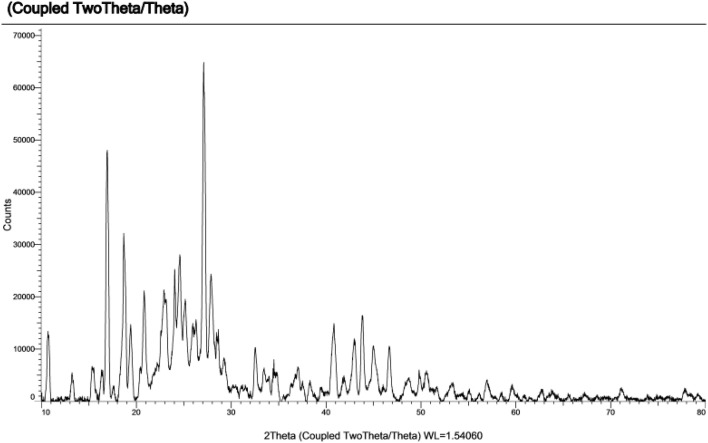
PXRD analysis of Cu–gallic acid MOF.

The PXRD pattern showed sharp peaks indicating the successful formation of the crystalline MOF structure (Fig. 5).

### 4.2. Lacasse-like activity of Cu–GA MOF

**Fig. 6:**
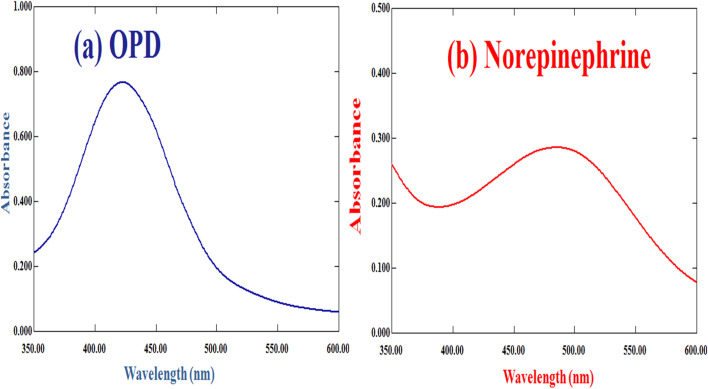
Spectral figure of (a) OPD and (b) norepinephrine.

The spectral analysis (Fig. 6) demonstrated a distinct determination of the substrates *o*-phenylenediamine and norepinephrine thereby substantiating their formation and functional properties and the successful application of Cu–GA MOF as an artificial enzyme.

### 4.3. Mimetic laccase activity of Cu–GA MOF

**Fig. 7:**
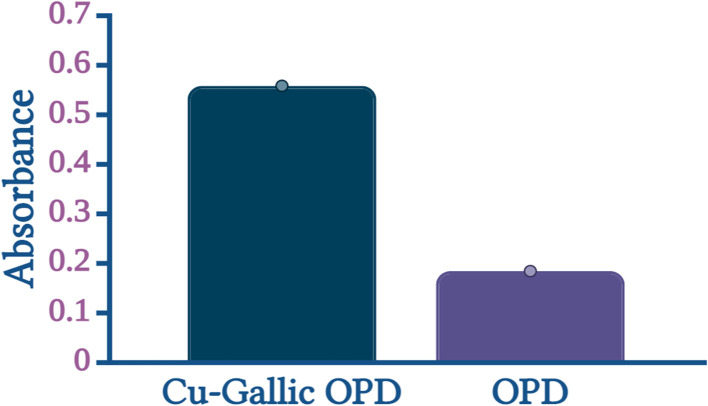
Investigation of catalytic activity of Cu–GA MOF on the oxidation of OPD.

The catalytic activity of Cu–GA MOF was evaluated by monitoring the absorbance of the oxidized product of *o*-phenylenediamine (OPD) under two experimental conditions (Fig. 7). In the first condition, which included both OPD and Cu–GA MOF significantly higher absorbance values were observed when measured using a UV-Vis spectrophotometer indicating a laccase-like catalytic activity. In contrast, under the second condition of the control experiment containing only OPD and without Cu–GA MOF negligible absorbance was observed demonstrating the minimal oxidation by oxygen under identical conditions. The increased absorbance in the presence of Cu–GA MOF strongly suggested that it facilitated the oxidation of OPD thereby exhibiting laccase-like catalytic properties.

### 4.4. Impact of pH on the catalytic performance of Cu–GA MOF

**Fig. 8:**
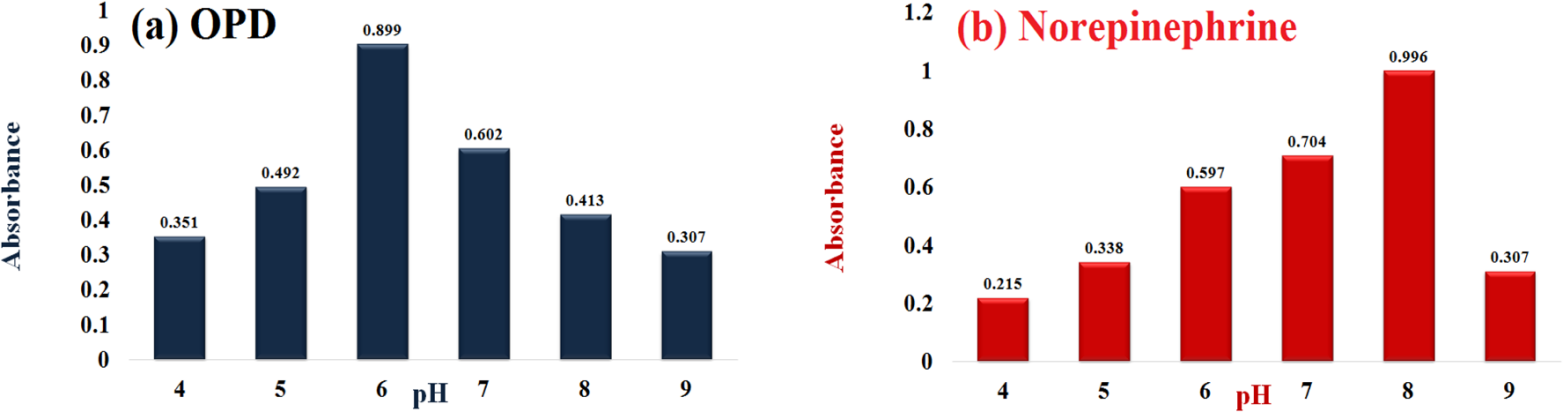
Influence of pH on the catalytic performance of Cu–GA MOF using (a) OPD and (b) norepinephrine as the substrates.

The catalytic activity of Cu–GA MOF was assessed across a pH range of 4.0–9.0 using *o*-phenylenediamine (OPD) and norepinephrine as the substrates. Results revealed a substrate-dependent pH sensitivity with optimal catalytic activity occurring at pH 6.0 for OPD and at pH 8.0 for norepinephrine (Fig. 8).

### 4.5. Impact of temperature on the catalytic performance of Cu–GA MOF

**Fig. 9:**
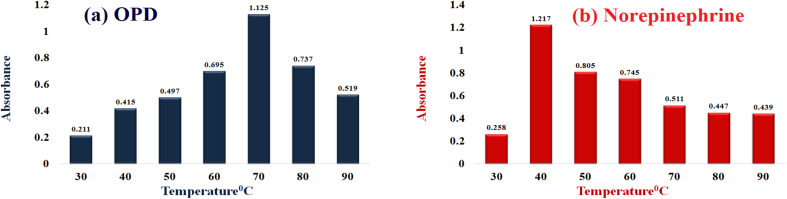
Influence of temperature on the catalytic performance of Cu–Ga MOF using (a) OPD and (b) norepinephrine as substrates.

The temperature dependence of the catalytic activity of Cu–GA MOF on *o*-phenylenediamine (OPD) and norepinephrine was evaluated across a temperature range of 30 °C to 90 °C. For OPD, a significant increase in absorbance was observed at 70 °C with the optimal catalytic activity occurring at this temperature. For norepinephrine, the highest catalytic activity was recorded between 40 °C and 50 °C. Both the OPD and norepinephrine reactions exhibited clear temperature-dependent patterns underscoring the importance of temperature optimization for optimal catalytic performance of Cu–GA MOF (Fig. 9).

### 4.6. Norepinephrine oxidation rate

**Fig. 10:**
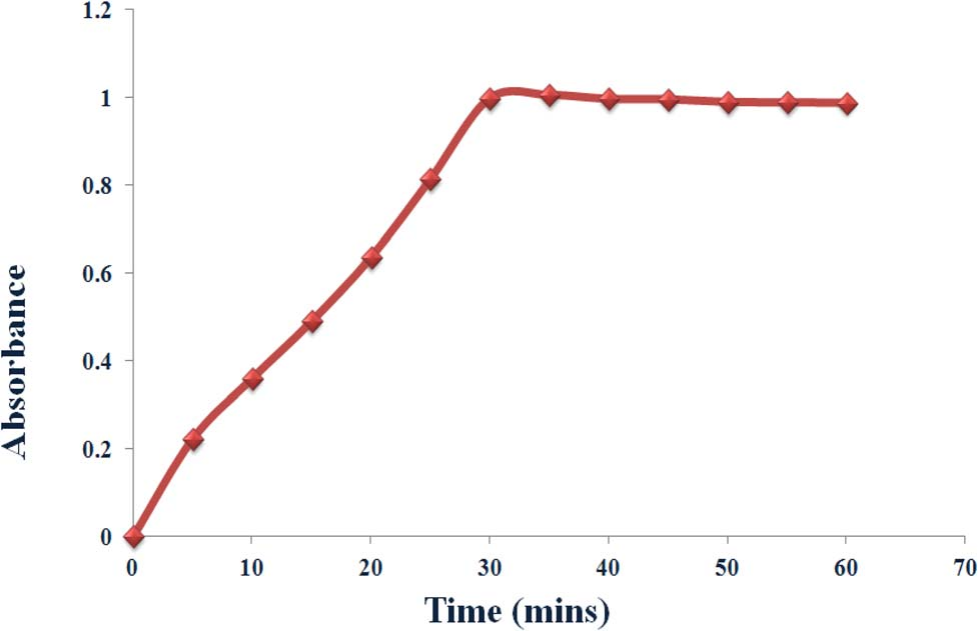
Norepinephrine oxidation rate by Cu–GA MOF.

**Scheme 1:**
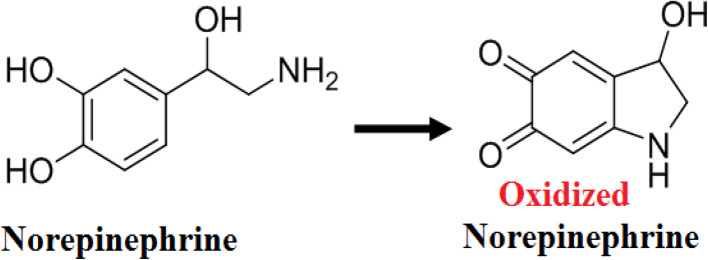
Proposed norepinephrine oxidation mechanism by Cu–GA MOF.

The oxidation rate of norepinephrine by Cu–GA MOF solution exhibited a time-dependent increase as determined by absorbance measurements at 5 min intervals over a 60 min reaction period using a UV-Vis spectrophotometer (Fig. 10). The reaction demonstrated an increase in the oxidation of norepinephrine as a function of time reaching its maximum between 30–35 minutes under the specified conditions (pH 8.0, 40 °C). Beyond this temperature, the reaction rate plateaued likely owing to substrate depletion or potential product inhibition. These findings indicate that Cu–GA MOF solution could serve as an efficient catalyst for the oxidative transformation of norepinephrine (Scheme 1).

### 4.7. Colorimetric determination of norepinephrine using Cu–GA MOF nanocatalyst

**Fig. 11:**
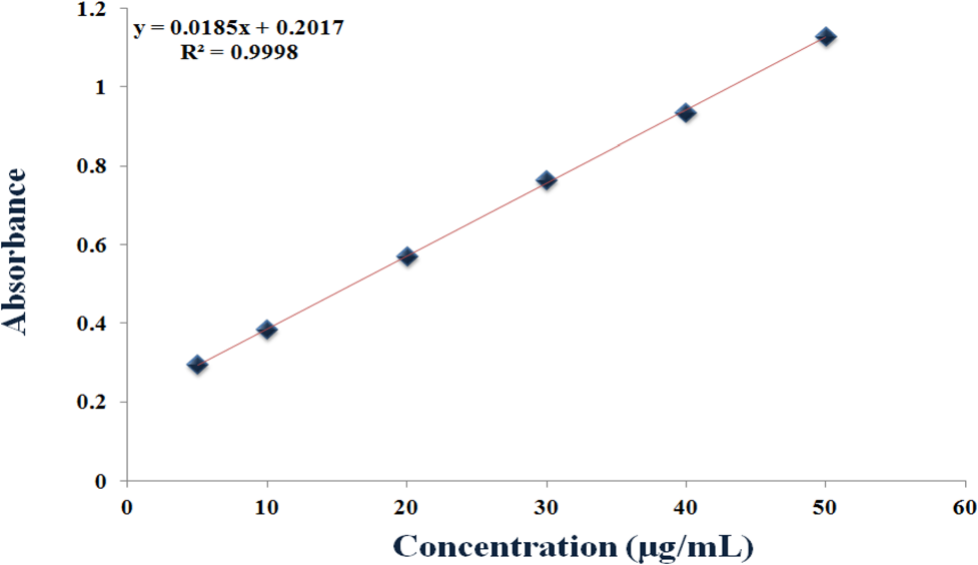
Calibration curve of norepinephrine catalyzed by Cu–GA MOF nanocatalyst.

The oxidation of norepinephrine catalyzed by the Cu–GA MOF solution exhibited a linear correlation between the norepinephrine concentration over a linear range of 5.0–50 μg mL^−1^ with an LOD of 4.11 and LOQ of 4.90 μg mL^−1^. The reaction demonstrated effective catalytic activity under the conditions of pH 8.0, 40 °C and 30 minutes of incubation time, with the absorbance increasing in direct proportion to the substrate concentration (Fig. 11). All the assay parameters were determined and showed acceptable results, as shown in Table 1.

**Table 1:** Assay parameters and method validation for the determination of norepinephrine utilizing Cu–GA MOF

Parameters	Norepinephrine
Linearity	5–50 μg mL^−1^

**Regression line**	
- Slope	0.0185
- Intercept	+0.2017
- Correlation coefficient (*r*)	0.9998
Accuracy (mean ± SD)^a^	101.95 ± 0.46

**Precision, (% RSD)**	
- Repeatability^b^	0.304
- Intermediate precision c	0.622
LOD^d^	4.11
LOQ^e^	4.90

a Average of three determinations (6, 25 and 45 μg mL^−1^).

b RSD%: the intra-day and (*n* = 3) relative standard deviations of concentrations (5, 30, and 50 μg mL^−1^).

c RSD%: the inter-day (*n* = 3) relative standard deviations of concentrations (5, 30, and 50 μg mL^−1^).

d Limit of detection (LOD) is calculated using the following equations: LOD = 3.3*σ*/*S*. Where “*σ*” is the mean of standard deviation of the intercept and “*S*” is the slope.

e Limit of quantification (LOQ) is calculated using the following equation: LOQ = 10*σ*/*S*. Where “*σ*” is the mean of standard deviation of the intercept and “*S*” is the slope.

### 4.8. Evaluation of the catalytic kinetics for norepinephrine oxidation by Cu–GA MOF

Results of catalytic kinetics evaluation demonstrated a substrate concentration-dependent increase in the reaction rate which was consistent with the Michaelis–Menten kinetics. As the norepinephrine concentration increased a linear rise in absorbance was observed indicating enhanced catalytic oxidation with the increase in substrate availability. The concentration study revealed that the catalytic activity of Cu–GA MOF on norepinephrine was directly proportional to the substrate concentration within the tested range.

**Fig. 12:**
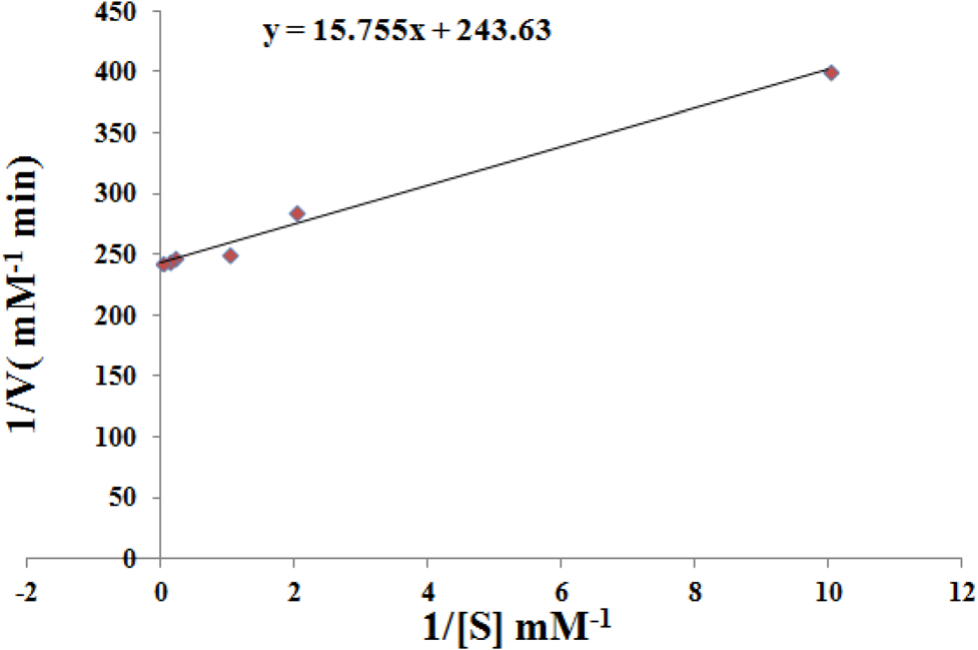
Lineweaver–Burk plot for the oxidization of norepinephrine by Cu–GA MOF.

Kinetics analysis was carried out by fixing the catalyst concentration at 100 μg mL^−1^ while the concentration of norepinephrine was varied between 0.1 mM and 50 mM. Absorbance measurements at 485.0 nm were quantitatively converted to the concentration of the norepinephrine oxidation product by applying the Beer–Lambert law. The Michaelis–Menten model was used to determine *K*_m_ and *V*_max_, with the final results presented in Fig. 12. Since the *K*_m_ value reflects the affinity of nanozymes for their substrates, the lower *K*_m_ value observed for Cu–GA MOF indicates a stronger interaction with norepinephrine compared to natural laccase as demonstrated by the comparative data presented; Table 2. Moreover, the *V*_max_ of Cu–GA MOF nanozyme was higher than that of the previously reported catalyst.^33^ This indicates that a lone particle of Cu–GA MOF nanozyme possesses a greater revenue rate, catalytic competence, and a superior active number of sites in comparison with laccase. The improvements in the kinetic parameters and catalytic performance suggest that mimicking the architectural structure of natural enzyme active sites can effectively improve nanozyme performance.

**Table 2:** Comparison of kinetic parameters of Cu–GA MOF with a previously reported catalyst

Catalyst	*K*_m_ (mM)	*V*_max_ (mM min^−1^)
Cu–gallic acid MOF	0.06	4.1 × 10^−3^
Copper-cysteine (C)–Aspartic (A) dipeptide CA-CU (reported work)	0.12	7.82 × 10^−3^

## 5. Conclusion

This study presents the successful synthesis and evaluation of a Cu–gallic acid metal–organic framework as a laccase-mimetic nanozyme. The findings emphasized that Cu–GA MOF exhibited pronounced catalytic activity in mediating the oxidation of phenolic substrates specifically *o*-phenylenediamine and norepinephrine. The catalytic performance was influenced by factors such as the pH, temperature, and substrate concentration. The nanozyme demonstrated optimal activity at pH 6.0 and 70 °C for OPD and at pH 8.0 and 40 °C for norepinephrine underscoring its potential for applications under mild environmental and biochemical conditions. The synthesized nanozyme offers a promising cost-effective alternative to natural laccases for environmental remediation and biosensing with high stability, recyclability, and catalytic efficiency. This research paves the way for the development of advanced nanozymes with enhanced laccase-like activity contributing to sustainable solutions in catalysis and environmental science.

## Data availability

All the data are provided within the article.

## Author contributions

Ola G. Hussein: conceptualization, methodology, validation, investigation, writing – original draft, visualization. Yara Mohamed: conceptualization, methodology, validation, investigation, visualization. Noha Mostafa: conceptualization, methodology, validation, investigation, visualization. Amr M. Mahmoud: conceptualization, methodology, validation, investigation, writing – original draft, visualization.

## Conflicts of interest

There are no conflicts to declare.

## Supplementary Material

RA-015-D5RA00942A-s001
