# Supplementary material for: Catalytic activity study of a laccase-like copper–gallic acid MOF and its applications in the colorimetric determination of norepinephrine and degradation of environmental pollutants
Source: RSC Adv. 2025 Apr 15;15(15):11730–8. doi: 10.1039/d5ra00942a (PMC11997649; doi:10.1039/d5ra00942a)
Supplement: RA-015-D5RA00942A-s001 [file RA-015-D5RA00942A-s001.pdf]

Supplementary Information (SI) for RSC Advances.
This journal is © The Royal Society of Chemistry 2025

# Supplementary Material

# For

# "Catalytic Activity Study of Laccase-like Copper-Gallic Acid; Colorimetric Determination of Norepinephrine and Degradation of Environmental Pollutant"

**Ola G. Hussein[1*], Yara Mohamed [2], Noha Mostafa[2], Amr M. Mahmoud[2,3]**

[1]*Department of Pharmaceutical Chemistry, Faculty of Pharmacy, Future University in Egypt, Cairo, 11835, Egypt*
[2]*Department of Chemistry, School of Pharmacy, Newgiza University, Km. 22 Cairo-Alex Road, Giza P.O. Box 12577, Egypt*
[3]*Department of Pharmaceutical Analytical Chemistry, Faculty of Pharmacy - Cairo University, Kasr El-Aini Street, ET-11562, Cairo - Egypt*

*Corresponding author email: ola.farag@fue.edu.eg

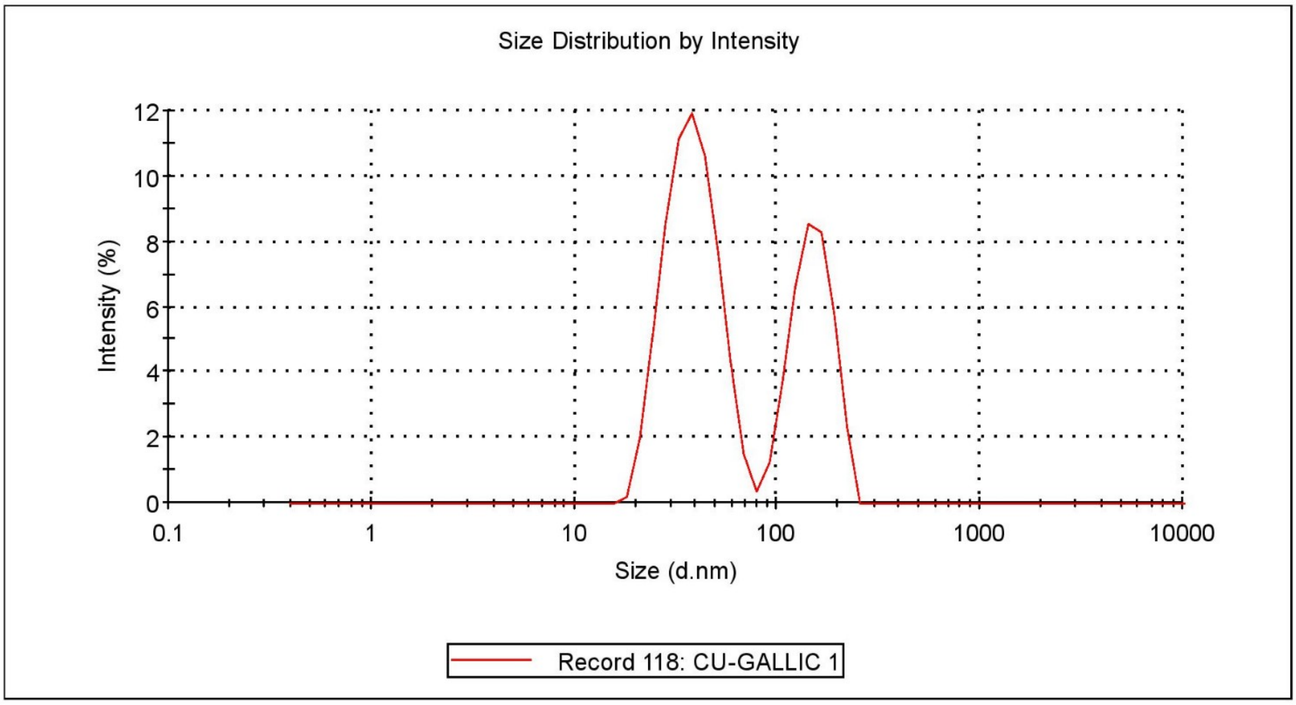


**Fig. S1 Cu-Gallic acid particle size using dynamic light scattering (DLS).**

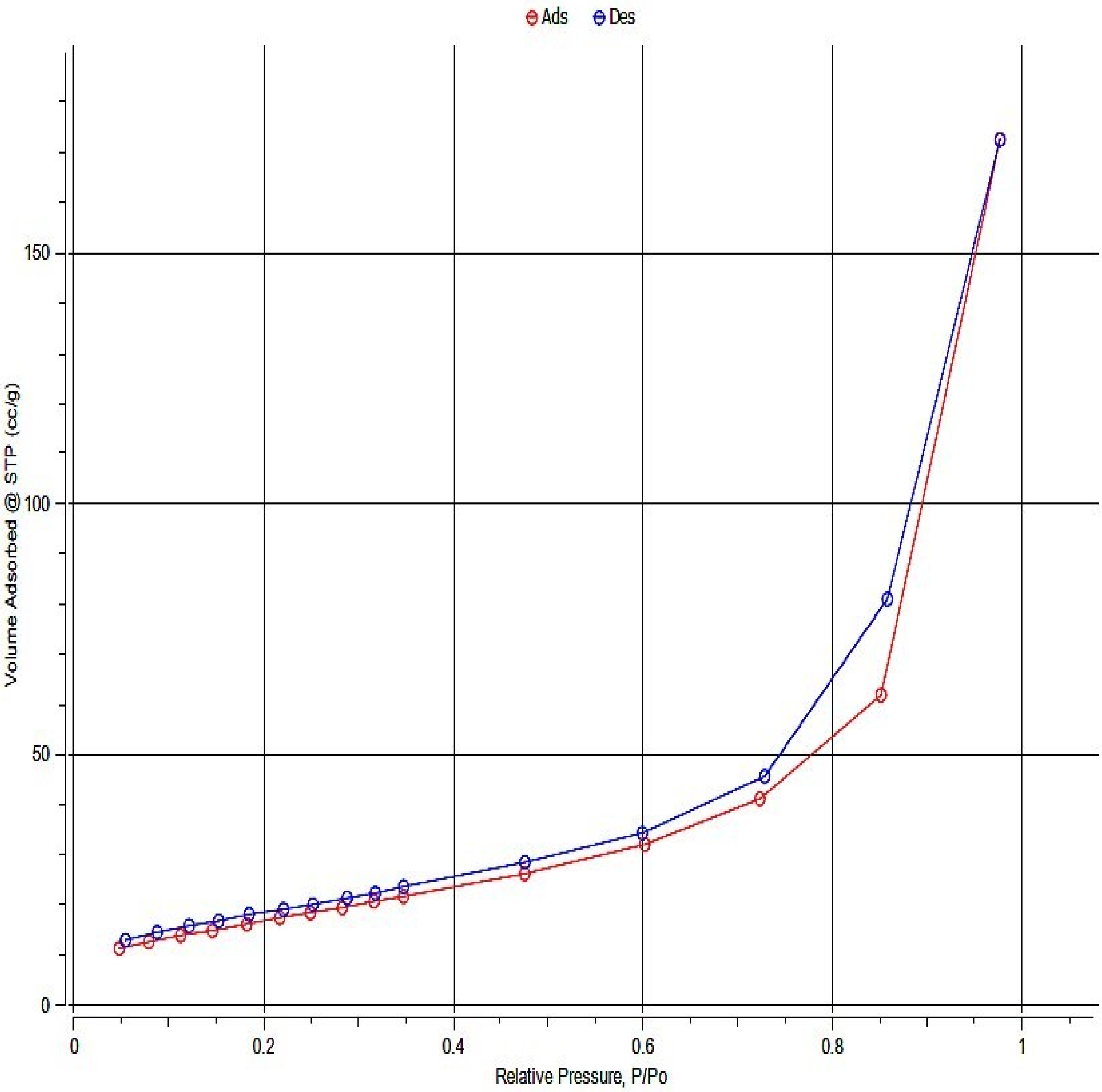


**Fig S2. Nitrogen Adsorption-Desorption Isotherm of the Prepared Cu-gallic acid MOF.**

**Table S1: Porosity and Surface Area Analysis of the Prepared Cu-gallic acid MOF**

| Method | Surface Area ($m^2/g$) | Pore Volume (cc/g) | Pore Radius (nm) |
|---|---|---|---|
| **BET (Multipoint)** | 1050.40 | - | - |
| **BET (Single-point)** | 1025.90 | - | - |
| **Langmuir Method** | 1480.30 | - | - |
| **BJH Adsorption** | 850.20 | 1.15 | 3.80 |
| **BJH Desorption** | 825.60 | 1.08 | 4.10 |
| **DH Adsorption** | 875.90 | 1.12 | 3.85 |
| **DH Desorption** | 840.50 | 1.05 | 4.00 |
| **DFT Method** | 1205.70 | 1.02 | 3.95 |
